# An early warning model for predicting major adverse kidney events within 30 days in sepsis patients

**DOI:** 10.3389/fmed.2023.1327036

**Published:** 2024-02-26

**Authors:** Xiaoyuan Yu, Qi Xin, Yun Hao, Jin Zhang, Tiantian Ma

**Affiliations:** ^1^Department of Hematology, The Affiliated Hospital of Northwest University, Xi’an No. 3 Hospital, Shaanxi, Xi’an, China; ^2^Department of Hepatobiliary Surgery, The First Affiliated Hospital of Xi’an Jiaotong University, Xi’an, Shaanxi, China; ^3^Department of Nephrology, Yuequn Yuan District, The First Hospital of Jilin University, Changchun, Jilin, China

**Keywords:** sepsis, major adverse kidney events within 30 days, nomogram model, early warning, MIMIC IV

## Abstract

**Background:**

In sepsis patients, kidney damage is among the most dangerous complications, with a high mortality rate. In addition, major adverse kidney events within 30 days (MAKE30) served as a comprehensive and unbiased clinical outcome measure for sepsis patients due to the recent shift toward targeting patient-centered renal outcomes in clinical research. However, the underlying predictive model for the prediction of MAKE30 in sepsis patients has not been reported in any study.

**Methods:**

A cohort of 2,849 sepsis patients from the Medical Information Mart for Intensive Care (MIMIC)-IV database was selected and subsequently allocated into a training set (*n* = 2,137, 75%) and a validation set (*n* = 712, 25%) through randomization. In addition, 142 sepsis patients from the Xi’An No. 3 Hospital as an external validation group. Univariate and multivariate logistic regression analyses were conducted to ascertain the independent predictors of MAKE30. Subsequently, a nomogram was developed utilizing these predictors, with an area under curve (AUC) above 0.6. The performance of nomogram was assessed through calibration curve, receiver operating characteristics (ROC) curve, and decision curve analysis (DCA). The secondary outcome was 30-day mortality, persistent renal dysfunction (PRD), and new renal replacement therapy (RRT). MAKE30 were a composite of death, PRD, new RRT.

**Results:**

The construction of the nomogram was based on several independent predictors (AUC above 0.6), including age, respiratory rate (RR), PaO2, lactate, and blood urea nitrogen (BUN). The predictive model demonstrated satisfactory discrimination for MAKE30, with an AUC of 0.740, 0.753, and 0.821 in the training, internal validation, and external validation cohorts, respectively. Furthermore, the simple prediction model exhibited superior predictive value compared to the SOFA model in both the training (AUC = 0.710) and validation (AUC = 0.692) cohorts. The nomogram demonstrated satisfactory calibration and clinical utility as evidenced by the calibration curve and DCA. Additionally, the predictive model exhibited excellent accuracy in forecasting 30-day mortality (AUC = 0.737), PRD (AUC = 0.639), and new RRT (AUC = 0.846) within the training dataset. Additionally, the model displayed predictive power for 30-day mortality (AUC = 0.765), PRD (AUC = 0.667), and new RRT (AUC = 0.783) in the validation set.

**Conclusion:**

The proposed nomogram holds the potential to estimate the risk of MAKE30 promptly and efficiently in sepsis patients within the initial 24 h of admission, thereby equipping healthcare professionals with valuable insights to facilitate personalized interventions.

## Introduction

Sepsis is defined as life-threatening organ dysfunction caused by a dysregulated host response to infection (1). It is a leading cause of mortality for patients, accounting for 25–30%, while septic shock is associated with a mortality rate of 45–63% (2). In addition, sepsis is particularly harmful to the kidney, and sepsis-induced acute kidney injury (S-AKI) is associated with a high mortality rate (3, 4). Furthermore, S-AKI is one of the suitable events for evaluating significant renal adverse events in a clinical setting, such as mortality and dependence on dialysis.

In 2012, the National Institute of Diabetes and Digestive and Kidney Diseases workgroup on clinical trials in Acute Kidney Injury (AKI) suggested the adoption of major adverse kidney events within 30 days (MAKE30) as an endpoint (5). MAKE30 encompasses the effects of both short-term and long-term progression of AKI, as it combines the occurrence of death, persistent renal dysfunction (PRD), or new renal replacement therapy (RRT) (5). Furthermore, MAKE30 served as a comprehensive and unbiased clinical outcome measure for sepsis patients due to the recent shift toward targeting patient-centered renal outcomes in clinical research. Consequently, the prediction of MAKE30 at an early stage and the subsequent personalized management could potentially augment the overall clinical prognosis.

The selection of appropriate endpoints in clinical trials assumes paramount significance, prompting researchers to recognize the significance of patient-centered outcomes, including mortality, dialysis, and the progression of chronic kidney disease, as pivotal factors for patients’ well-being. MAKE30 has been employed as a suitable test endpoint in various clinical trials, such as Saline Against Lactated Ringer’s or Plasma-Lyte (SALT) (6), Isotonic Solutions and Major Adverse Renal Events Trial (SMART) (7), and pediatric sepsis trials (8). By utilizing MAKE30, it becomes feasible to detect short-term effects on AKI, and facilitate the conduct of clinical trials. Nevertheless, there are still no reliable and robust predictors to identify high-risk patients likely to develop MAKE30. For instance, serum creatinine and urine volume are utilized as predictors of AKI. Nevertheless, these markers exhibit variation until 50% of renal function is compromised, suggesting their limited relevance to AKI. Furthermore, when employed as individual indices, these markers often lack sensitivity or specificity. Hence, the construction of a nomogram that combines these factors holds significant clinical significance in identifying the occurrence of MAKE30 in sepsis patients. In this study, MAKE30 is a composite of death, receipt of new RRT, or PRD censored at hospital discharge or 30 days after inclusion, whichever occurred first. Our study aimed to identify the risk factors, obtainable within 24 h of intensive care unit (ICU) admission, for MAKE30 development in sepsis patients, with the objective of constructing an early prediction model to identify individuals at high risk.

## Materials and methods

### Study design

The data of this retrospective cohort study were extracted from the Medical Information Mart for Intensive Care (MIMIC)-IV, a famous clinical critical care database containing 730,141 admissions information from 2008 to 2019. Qi Xin, one of the authors, collected clinical data from the MIMIC database via an exam called Protection Human Research Participants (certification number: 53163698). Furthermore, a retrospective cohort study of 142 sepsis patients was conducted from January 2023 to December 2023, and anonymized clinical data were taken from the data processing and application platform of the Xi’An No. 3 Hospital.

### Patients

All patients aged 18 years and older with sepsis were evaluated for study enrollment. According to the Sepsis 3 criteria, sepsis was defined as documented or suspected infection with sequential organ failure assessment (SOFA) score ≥ 2 (1). The following were the exclusion criteria: (1) <18 years old; (2) The length of stay in ICU was less than 24 h; (3) Multiple ICU admission patients; (4) End-stage renal disease; (5) Hematological disorders; (6) Missing information, such as some laboratory test indicators. Based on the diagnostic criteria of MAKE30, patients were divided into two groups: the MAKE30 group and the non-MAKE30 group (9). The diagnosis of MAKE30 was based on any one of the following criteria: (1) mortality; (2) initiation of RRT for the initial occasion; (3) the occurrence of a PRD. PRD is characterized by a final inpatient serum creatinine level censored at hospital discharge equal to or exceeding 200% of the baseline value. In addition, the baseline level of serum creatinine was determined as the lowest value recorded within the period of 12 months to 24 h prior to hospitalization (9). These three components of MAKE30 were censored 30 days after inclusion or at hospital release, whichever occurred first.

### Data collection

Data were extracted from MIMIC-IV: (1) the general information included age, gender, temperature, heart rate (HR), RR, (mean arterial pressure) MAP, SOFA, and comorbidities available within 24 h after ICU admission; (2) laboratory tests within 24 h of ICU admission included white blood cells (WBC), neutrophil percentage (NEUT%), lymphocyte, monocyte, platelet (PLT), activated partial thromboplastin Time (APTT), prothrombin time (PT), PH, base excess (BE), actual bicarbonate (AB), Total CO2, PaO2, PaCO2, lactate, blood urea nitrogen (BUN), and creatinine (Cr); (3) final inpatient serum Cr for assessing PRD;(4) outcome data included mortality, PRD, new RRT, ICU length of stay (LOS), Hospital LOS.

With 5-10 potential modeling predictors reported in previous studies and considering the 20% MAKE30 composite outcome from preliminary investigation, we estimated that 250-500 patients (with 50-100 MAKE30 composite outcome) were needed for sufficient precision of model construction to follow the principle of at least ten outcome events per variable in the regression analysis.

### Statistical analysis

The construction of the prediction model involved using the training group (75% of the sample size), while the accuracy of the model was verified using the validation group (25% of the sample size). Continuous variables that followed a normal distribution were expressed as mean ± standard deviation, while non-normally distributed variables were expressed as median (interquartile range). Categorical variables were expressed as the percentages. Univariate and multivariate logistic regression models were used to further determine the independent predictors of MAKE30. Based on a widely acknowledged standard, an area under curve (AUC) value below 0.6 is deemed indicative of low prediction, while a range of 0.6–0.75 signifies medium prediction, and a value exceeding 0.75 indicates high prediction (10). The nomogram was constructed using the independent predictors with an AUC above 0.6. Furthermore, we constructed an interactive and accessible online nomogram using the “DynNom” package. We used receiver operating characteristics (ROC) curves to assess the precision of the nomogram for MAKE30, calibration curves to evaluate the consistency of the observed results and predicted probability, and decision curve analysis (DCA) to assess the clinical net benefit of the predictive model. In addition, ROC curves were used to assess the prediction of the nomogram for 30-day mortality, PRD, and RRT in subgroup analyses.

The analyses were conducted using SPSS 26.0 and R version 4.1.2, and *P* < 0.05 was considered statistically significant.

## Results

### Basic characteristics

**FIGURE 1:**
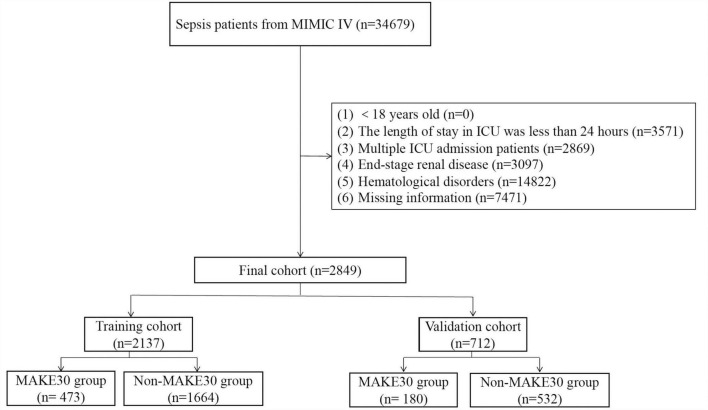
The flowchart of patient selection from the Medical Information Mart for Intensive Care (MIMIC)-IV.

As shown in Figure 1, 2,849 patients with sepsis were selected in the final analyses, of whom 653 (22.9%) reached MAKE30. We excluded patients with hematologic disorders, nearly half of the sepsis patients from MIMIC IV. Due to immune system disorders, patients with hematologic disorders are more likely to be attacked by bacteria, resulting in sepsis. However, laboratory test indicators of patients with hematologic disorders are disturbed and cannot truly reflect the severity of sepsis. Furthermore, the training group (75%, *n* = 2,137) and validation group (25%, *n* = 712) were selected randomly from the total cases. Similar demographic characteristics, vital signs, comorbidities, laboratory examinations and outcome data between the training and validation sets are shown in Table 1. In this study, 22.1 and 25.3% of sepsis patients reached MAKE30 in the training and validation groups, respectively. Moreover, the median days of death and RRT were 5.34 and 2.28 days, respectively. The median age of participants was 67.5 years and 67.7 years, with 39.4 and 39.9% of patients being female in the training and validation sets, respectively.

**TABLE 1:** Baseline characteristics of the patients with sepsis.

Variable	Total (*n* = 2,849)	Training (*n* = 2,137)	Validation (*n* = 712)	*P-*value
MAKE30 [n (%)]				0.084
No	2,196 (77.1)	1,664 (77.8)	532 (74.7)	
Yes	653 (22.9)	473 (22.1)	180 (25.3)	
Morality [n (%)]				0.152
No	2,263 (79.4)	1,711 (80.1)	552 (77.5)	
Yes	586 (20.6)	426 (19.9)	160 (22.5)	
PRD [n (%)]				0.458
No	2,742 (96.2)	2,060 (96.4)	682 (95.8)	
Yes	107 (3.8)	77 (3.6)	30 (4.2)	
RRT [n (%)]				0.493
No	2,760 (96.8)	2,073 (97)	687 (96.5)	
Yes	89 (3.2)	64 (3)	25 (3.5)	
Age	67.6 (56.6, 78.5)	67.5 (56.6, 78.3)	67.7 (56.4, 78.8)	0.862
Gender [n (%)]				0.801
Female	1,125 (39.5)	841 (39.4)	284 (39.9)	
Male	1,724 (60.5)	1,296 (60.6)	428 (60.1)	
Vital signs				
T (°C)	36.7 (36.2, 37.1)	36.7 (36.2, 37.1)	36.7 (36.2, 37.1)	0.972
RR (bpm)	18 (15, 23)	18 (15, 23)	19 (16, 24)	0.077
HR (bpm)	88 (77, 104)	88 (76, 103)	88 (78, 105)	0.329
MAP (mmHg)	77 (67, 91)	77 (67, 91)	67 (76, 91)	0.858
Comorbidities				
Hypertension [n (%)]				
No	1,016 (35.7)	765 (35.8)	251 (35.3)	0.793
Yes	1,833 (64.3)	1,372 (64.2)	461 (64.7)	
Diabetes [n (%)]				
No	1,951 (69.0)	1,448 (67.8)	503 (70.6)	0.151
Yes	898 (31.0)	689 (32.2)	209 (29.4)	
Cardiovascular disease [n (%)]				
No	1,756 (61.6)	1,318 (61.7)	438 (61.5)	0.940
Yes	1,093 (38.4)	819 (38.3)	274 (38.5)	
Laboratory test				
WBC (x10^9^/L)	12.1 (8.7, 16.6)	12.0 (8.7, 16.7)	12.2 (8.5, 16.4)	0.555
Monocyte (x 10^9^/L)	0.48 (0.29, 0.77)	0.47 (0.28, 0.77)	0.49 (0.30, 0.78)	0.628
NEUT (%)	82.2 (74.0, 88.0)	82.3 (74.1, 88.0)	82.0 (73.8, 88.0)	0.826
Lymphocyte (x 10^9^/L)	1.17 (0.74, 1.80)	1.18 (0.75, 1.81)	1.16 (0.70, 1.77)	0.315
PLT (x 10^9^/L)	216 (163, 286)	216 (163, 285)	219 (164, 290)	0.377
APTT (S)	29.6 (26.3, 34.8)	29.6 (26.3, 34.8)	29.8 (26.3, 35.1)	0.372
PT (S)	13.9 (12.3, 16.2)	13.9 (12.4, 16.2)	14.0 (12.3, 16.2)	0.832
PH	7.37 (7.3, 7.42)	7.37 (7.30, 7.42)	7.37 (7.30, 7.42)	0.956
BE (mmol/L)	0 (−4, 2)	0 (−4, 2)	0 (−4, 2)	0.699
AB (mmol/L)	23 (20, 26)	23 (20, 26)	23 (20, 26)	0.343
Total CO2 (mmHg)	25 (22, 28)	25 (22, 28)	25 (21, 28)	0.534
PaO2 (mmHg)	152 (88, 312)	155 (87, 317)	143 (88, 299)	0.274
PaCO2 (mmHg)	41 (36, 49)	42 (36, 49)	41 (36, 48)	0.364
Lactate (mmol/L)	1.5 (1.1, 2.4)	1.5 (1.1, 2.4)	1.6 (1.1, 2.3)	0.979
BUN (mg/dl)	20 (14, 30)	20 (14, 30)	20 (14, 31)	0.898
Cr (mg/dl)	1.0 (0.8, 1.4)	1.0 (0.8, 1.5)	1.0 (0.8, 1.4)	0.772
SOFA	5 (4, 8)	5 (4, 8)	5 (4, 8)	0.978
ICU LOS (days)	3.3 (1.8, 6.4)	3.2 (1.8, 6.6)	3.3 (2.0, 6.1)	0.400
Hospital LOS (days)	7.9 (5.0, 13.0)	7.8 (4.9, 13.1)	8.1 (5.0, 12.7)	0.986

MAKE30, major adverse kidney events within 30 days; RRT, renal replacement therapy; PRD, persistent renal dysfunction; T, body temperature; HR, heart rate; RR, respiratory rate; MAP, mean arterial pressure; WBC, white blood cell; NEUT%, neutrophil percentage; PLT, platelet; APTT, activated partial thromboplastin time; PT, prothrombin time; BE, base excess; AB, actual bicarbonate; BUN, blood urea nitrogen; Cr, creatinine; SOFA, sequential organ failure assessment; ICU, intensive care unit; LOS, Length of stay.

### Univariate analysis results

**FIGURE 2:**
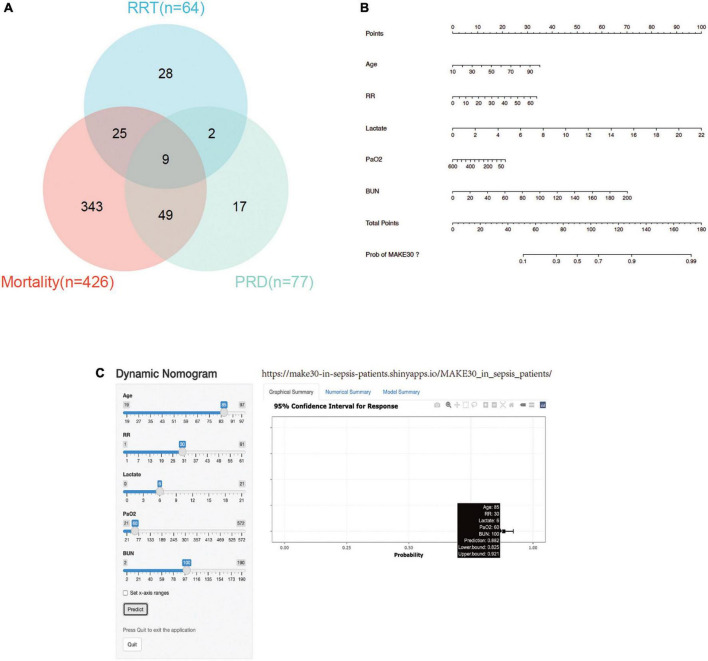
MAKE30 in the training set. **(A)** Venn diagram of MAKE30 components in the training set; **(B)** Nomogram to estimate the risk of MAKE30 in sepsis patients; **(C)** Online version of this nomogram to estimate the risk of MAKE30 dynamically. MAKE30, major adverse kidney events within 30 days; RRT, renal replacement therapy; PRD, persistent renal dysfunction; RR: respiratory rate; BUN: blood urea nitrogen.

In the training cohort, the mortality incidence for the individual components of MAKE30, as depicted in Venn diagram (Figure 2A), was 426 (19.9%), with new RRT at 64 (3.0%) and PRD at 77 (3.6%). Univariate analyses in Table 2 revealed that age (1.027, 1.019–1.034), RR (1.053, 1.036–1.069), HR, WBC, Monocyte, PLT, APTT, PT, lactate (1.315, 1.241–1.394), BUN (1.027, 1.022–1.032), and Cr significantly increased in the MAKE30 group than in the non-MAKE30 group. While temperature, PH, BE, AB, Total CO2, and PaO2 (0.997, 0.996–0.998) were significantly lower in the MAKE30 group. In addition, SOFA and ICU LOS were significantly increased in the MAKE30 group.

**TABLE 2:** Univariate analysis of predictive variables of MAKE30 in the training cohort.

Variables	OR	95% CI	*P-*value
Age (years)	1.027	1.019–1.034	<0.001
Gender	0.818	0.665–1.006	0.057
**Vital signs**			
T (°C)	0.883	0.798–0.978	0.017
RR (bpm)	1.053	1.036–1.069	<0.001
HR (bpm)	1.013	1.008–1.018	<0.001
MAP (mmHg)	1.003	0.997–1.008	0.315
**Comorbidities**			
Hypertension	0.939	0.758–1.163	0.563
Diabetes	0.981	0.789–1.222	0.867
Cardiovascular disease	0.877	0.710–1.085	0.227
**Laboratory test**			
WBC (x10^9^/L)	1.034	1.020–1.049	<0.001
Monocyte (x 10^9^/L)	1.423	1.162–1.743	0.001
NEUT (%)	1.008	1.000–1.017	0.056
Lymphocyte (x 10^9^/L)	1.012	0.991–1.033	0.257
PLT (x 10^9^/L)	1.001	1.000–1.002	0.004
APTT (S)	1.008	1.003–1.013	0.002
PT (S)	1.020	1.011–1.029	<0.001
PH	0.047	0.018–0.122	<0.001
BE (mmol/L)	0.935	0.918–0.953	<0.001
AB (mmol/L)	0.952	0.934–0.971	<0.001
Total CO2 (mmHg)	0.955	0.938–0.972	<0.001
PaO2 (mmHg)	0.997	0.996–0.998	<0.001
PaCO2 (mmHg)	1.001	0.995–1.008	0.712
Lactate (mmol/L)	1.315	1.241–1.394	<0.001
BUN (mg/dl)	1.027	1.022–1.032	<0.001
Cr (mg/dl)	1.467	1.336–1.612	<0.001
SOFA	1.298	1.253–1.344	<0.001
ICU LOS (days)	1.024	1.007–1.041	0.006
Hospital LOS (days)	0.952	0.937–0.968	<0.001

T, body temperature; HR, heart rate; RR, respiratory rate; MAP, mean arterial pressure; WBC, white blood cell; NEUT%, neutrophil percentage; PLT, platelet; APTT, activated partial thromboplastin time; PT, prothrombin time; BE, base excess; AB, actual bicarbonate; BUN, blood urea nitrogen; Cr, creatinine; SOFA, sequential organ failure assessment; ICU, intensive care unit, LOS, Length of stay.

### Predictors of MAKE30 and nomogram development

Multivariate logistic regression analyses were performed using the significant different variables, including age, temperature, RR, HR, WBC, Monocyte, PLT, APTT, PT, PH, BE, AB, Total CO2, PaO2, lactate, BUN, and Cr. As depicted in Table 3, age, temperature, RR, HR, WBC, PaO2, lactate, and BUN were independent predictors for MAKE30 in sepsis patients. Furthermore, a nomogram based on these traits (with an AUC value above 0.6) was created to predict MAKE30 in sepsis patients (Table 4; Figure 2B). In addition, we used the “DynNom” package to provide an online version of this nomogram^1^ to facilitate the use of this nomogram (Figure 2C).

**TABLE 3:** Multivariate logistic regression analysis of independent predictors of MAKE30 in the training cohort.

Variables	β	SE	Wald	*P*-value	OR (95% CI)
Age (years)	0.023	0.004	35.151	<0.001	1.023 (1.015–1.013)
T (°C)	-0.153	0.059	6.788	0.009	0.858 (0.765–0.963)
HR (bpm)	0.007	0.003	6.395	0.011	1.007 (1.002–1.013)
RR (bpm)	0.022	0.009	5.663	0.017	1.023 (1.009–1.038)
WBC (x10^9^/L)	0.023	0.007	10.246	0.001	1.023 (1.009–1.038)
PaO2 (mmHg)	-0.002	0	14.125	<0.001	0.098 (0.997, 0.999)
Lactate (mmol/L)	0.232	0.031	56.663	<0.001	1.262 (1.188, 1.340)
BUN (mg/dl)	0.019	0.003	50.344	<0.001	1.019 (1.014, 1.024)
Constant	0.607	2.144	0.080	0.777	–

T, body temperature; HR, heart rate; RR, respiratory rate; WBC, white blood cell; BUN, blood urea nitrogen.

**TABLE 4:** The AUC of independent predictors for the prediction of MAKE30 in the training cohort.

Variables	AUC	95% CI	*P-*value
Age (years)	0.622	0.592–0.652	<0.001
T (°C)	0.523	0.493–0.554	0.122
HR (bpm)	0.580	0.549–0.610	<0.001
RR (bpm)	0.608	0.580–0.636	<0.001
WBC (x10^9^/L)	0.573	0.542–0.603	<0.001
PaO2 (mmHg)	0.605	0.578–0.633	<0.001
Lactate (mmol/L)	0.640	0.611–0.669	<0.001
BUN (mg/dl)	0.661	0.632–0.690	<0.001

T, body temperature; HR, heart rate; RR, respiratory rate; WBC, white blood cell; BUN, blood urea nitrogen.

### Verification of the prediction model

**FIGURE 3:**
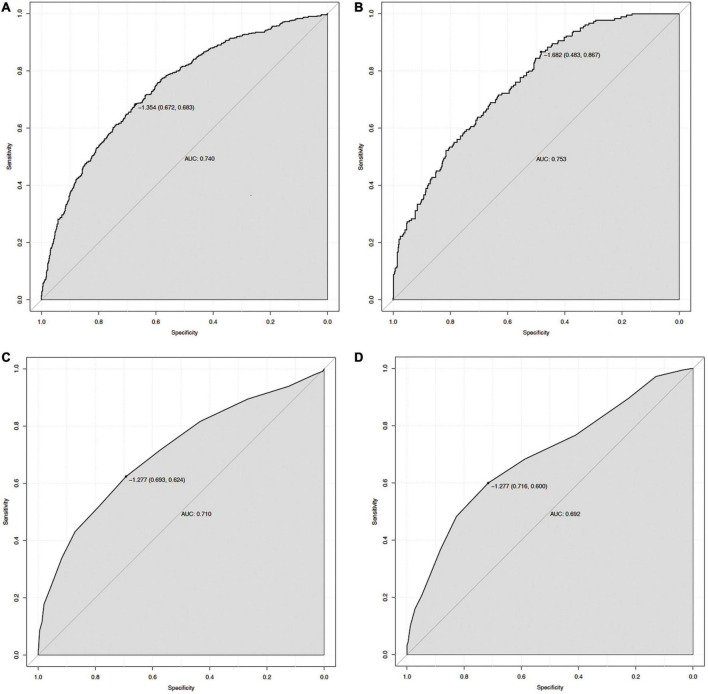
The ROC curve of the nomogram for predicting MAKE30 in sepsis patients. The AUC of the nomogram for the prediction of MAKE30 in sepsis patients was 0.740 in the training set **(A)** and 0.753 in the validation set **(B)**. The AUC of SOFA for the prediction of MAKE30 in sepsis patients was 0.710 in the training set **(C)** and 0.692 in the validation set **(D)**. ROC, receiver operating characteristic; AUC, area under the receiver operating characteristics curve; SOFA, sequential organ failure assessment.

**FIGURE 4:**
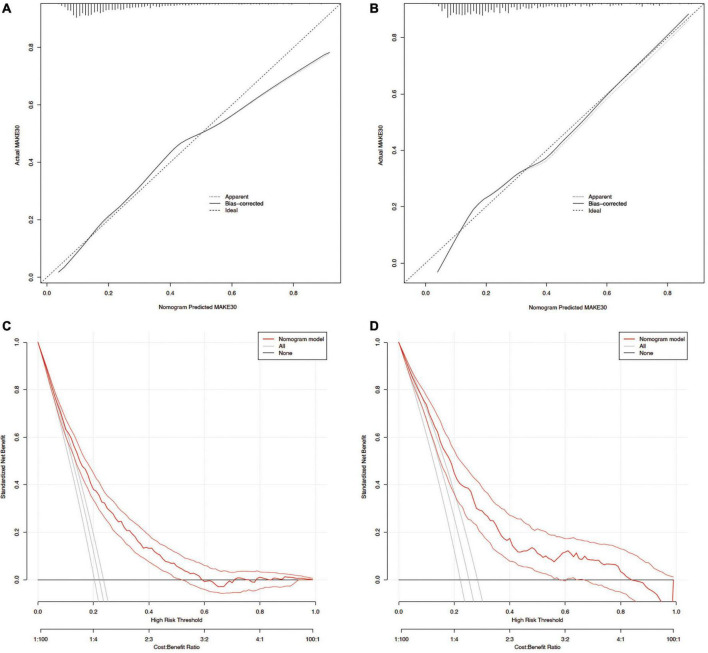
Calibration curves and DCA of the nomogram for predicting MAKE30. Calibration curves of the predicted nomogram in the training set **(A)** and validation set **(B)**; DCA of the nomogram in the training set **(C)** and the validation set **(D)**. DCA, Decision curve analysis.

The ROC curve analyses demonstrated that the nomogram had a good ability to predict MAKE30 from sepsis patients in the training (AUC = 0.740) and validation (AUC = 0.753) cohorts (Figure 3). Moreover, the simple prediction model had a better predictive value than SOFA in the training (AUC = 0.710) and validation (AUC = 0.692) cohorts (Figure 3). The calibration curve revealed that the predictive probabilities were in consistent agreement with the observation results in the training and validation cohorts, indicating a successful calibration (Figure 4). Based on the DCA, the nomogram had superior overall net benefit within the wide range of threshold probabilities, indicating high potential for clinical utility (Figure 4).

### The subgroup analyses using the prediction model

**FIGURE 5:**
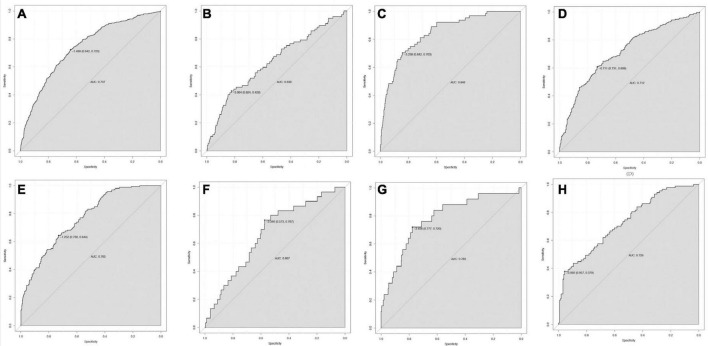
The ROC curve of the nomogram for predicting 30-day mortality **(A)**, PRD **(B)**, and RRT **(C)** in the training set; 30-day mortality **(E)**, PRD **(F)**, and new RRT **(G)** in the validation set. In patients with lactate ≥ 2 mmol/L, the ROC curve of the nomogram for predicting MAKE30 in the training set **(D)** and validation set **(H)**. RRT, renal replacement therapy; PRD, persistent renal dysfunction; ROC, Receiver operating characteristic; AUC, area under the receiver operating characteristics curve.

According to the findings presented in Figure 5, the ROC curve analyses demonstrated that the nomogram model exhibited significant predictive capability in forecasting 30-day mortality (AUC = 0.737), PRD (AUC = 0.639), and new RRT (AUC = 0.846) within the training set. Additionally, the model displayed predictive power for 30-day mortality (AUC = 0.765), PRD (AUC = 0.667), and new RRT (AUC = 0.783) in the validation set. In this study, 740 (34.6%) and 248 (34.8%) of sepsis patients reached lactate ≥ 2 mmol/L in the training and validation groups, respectively. In patients with lactate ≥ 2 mmol/L, the nomogram model also displayed predictive power for MAKE30 in the training (AUC = 0.712) and validation (AUC = 0.729) cohorts.

### The external validation using the prediction model

**FIGURE 6:**
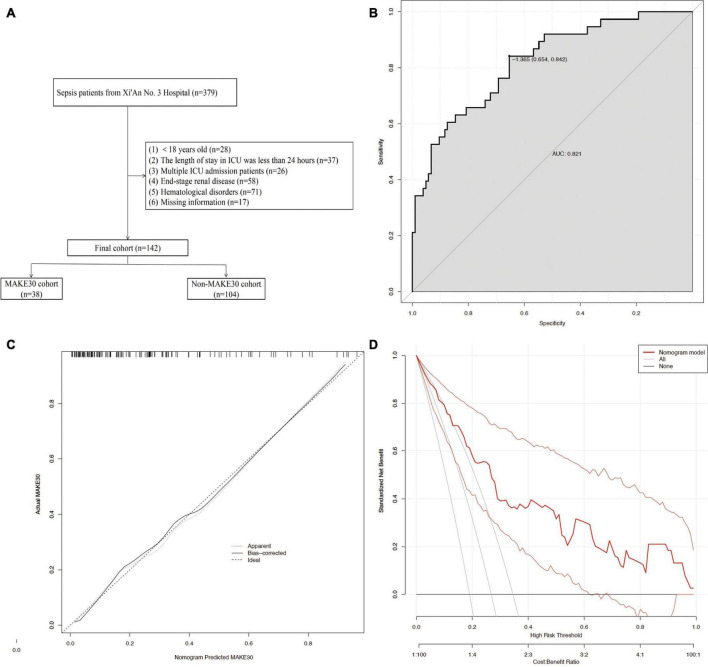
The external validation using the Prediction Model. The flowchart of patient selection **(A)** from the Xi’An No. 3 Hospital. The ROC curve **(B)**, Calibration curves **(C)**, and DCA **(D)** of the nomogram for predicting MAKE30 in the external validation set.

As shown in Figure 6, external validation of 142 sepsis patients from the Xi’An No. 3 Hospital showed that the nomogram was good at predicting MAKE30 (AUC = 0.821), with a specificity of 65.4% and a sensitivity of 84.2%. The calibration curve indicated a successful calibration and the DCA indicated high potential for clinical utility.

## Discussion

We developed a straightforward nomogram model to forecast MAKE30 in sepsis patients based on independent predictors (with an AUC value above 0.6), including age, RR, lactate, PaO2, and BUN within 24 h of admission. We collected more significant variables, such as laboratory data, available within 24 h after ICU admission to construct the nomogram model than previous studies (11). This study has revealed that out of the 653 (22.9%) sepsis patients examined, a MAKE30 composite outcome was observed, which is lower than the findings of a previous study (28.3%) conducted on sepsis patients, but higher than the outcome observed in patients with acute pancreatitis (16%) (9, 12). Of utmost significance is the model’s efficacy and ease of implementation. Furthermore, accurately predicting MAKE30 may facilitate both patient care and study of interventions. Recognition of patients at high risk for renal outcomes within 24 h after ICU admission could allow providers to avoid risk factors, such as nephrotoxic drugs and radiocontrast dye.

Classical theories propose that the elderly have high mortality, PRD, and RRT in sepsis patients because various factors may contribute to the decline in renal function with advancing age, encompassing the atrophy and degeneration of renal tubular epithelial cells, a thickened basement membrane, and reduced renal blood flow (13). In line with our study, previous research findings have demonstrated a substantial rise in the incidence of sepsis (58–65%) among elderly patients, with age being identified as an independent predictor of sepsis mortality (14). Furthermore, various studies have reported a progressive increase in sepsis mortality rates, with percentages rising from 10% in children to 26% in patients aged 60–64 and 38% in individuals over 85 (14). Additionally, the elderly are the primary age group affected by sepsis and vulnerable to developing MAKE30.

Renal hypotension, ischemia, and inadequate oxygen supply are the principal pathological mechanisms underlying AKI (4). Our study revealed that PaO2 and RR were independent predictors for the prediction of MAKE30. Oxygen supplementation is widely employed as an intervention in the treatment of critically ill individuals. Furthermore, numerous studies have demonstrated that a high partial pressure of oxygen in arterial blood (PaO2) effectively diminishes the incidence of poor prognosis in sepsis patients (15–18). Martín-Fernández et al. discovered that maintaining oxygenation levels with a PaO2 above 100 mmHg exhibited a significant correlation with reduced 90-day mortality, decreased duration of ICU stays, and shorter intubation time among critically ill patients with postsurgical sepsis/septic shock (19). The bactericidal activity of neutrophils is facilitated by oxidative killing, a vital defense mechanism against pathogens (20). This robust bactericidal mechanism relies on the generation of superoxide radicals from molecular oxygen (21). Analogous to the PaO2, RR also serves as an indicator of the body’s oxygen supply and the presence of hypoxia, both of which are factors associated with critical illness. Huabbangyang et al. conducted a study that revealed a correlation between higher RR and lower oxygen saturations with an increased 30-day mortality rate among sepsis patients (18). Consequently, both PaO2 and RR hold significant predictive value for the occurrence of MAKE30, and the administration of oxygen to achieve a higher PaO2 level may effectively reduce the likelihood of adverse renal outcomes in sepsis patients.

Serum lactate levels exceeding 2 mmol/L are recommended in sepsis 3 as the primary parameter for distinguishing septic shock clinically (1). Elevated plasma lactate levels observed in septic patients may arise from inadequate oxygen supply, mitochondrial impairment, and heightened glycolysis. Prior investigations have demonstrated a connection between early lactate levels and organ dysfunction as well as mortality in both the intensive care unit and emergency department, with elevated lactate levels exhibiting a positive correlation with heightened mortality rates (15, 22–24). The accumulation of lactate in the extracellular fluid leads to the depletion of bicarbonate anions (HCO3-) and the accumulation of lactate anions (C3H5O3-), thereby exacerbating lactic acidosis and sepsis (25). Additionally, it has been reported that fatty acid oxidation and the tricarboxylic acid cycle (TCA) are inhibited, while glycolysis is enhanced, resulting in excessive lactate production in renal tubular epithelial cells during AKI (26, 27). Furthermore, lactate is also implicated in lactylation processes (25). An et al. discovered that hyperacetylation and subsequent inactivation of the pyruvate dehydrogenase E1 component subunit alpha (PDHA1) lead to an increase in lactate production, which in turn promotes the lactylation of the mitochondrial fission 1 protein (Fis1) and worsens the severity of S-AKI (28). Conversely, decreasing lactate levels and Fis1 lactylation can alleviate S-AKI (28). Additionally, our findings revealed a significant association between lactate and MAKE30 in sepsis patients, underscoring the importance of therapeutic interventions aimed at reducing blood lactate levels in septic patients.

Blood urea nitrogen is the main metabolic waste product of protein in the liver and is excreted mainly by the kidney. Hence, BUN levels will increase when the rate of protein catabolism increases significantly during sepsis or when the glomerular filtration rate decreases in patients with AKI (29). Nevertheless, serum creatinine is slightly elevated by muscle proteolysis in critically ill patients. Consequently, BUN is more valuable in predicting S-AKI than serum creatinine because the rise in BUN is more pronounced than that of serum creatinine (30). Compared with serum creatinine, BUN is more sensitive to reflecting decreased renal perfusion, systemic hypovolemia, or decreased cardiac output (31). In addition, BUN has been demonstrated to be a strong indicator of prognosis, including decompensated heart failure, acute myocardial infarction, aortic dissection, acute pancreatitis, and general critical illness (32). Previous studies revealed that BUN is an independent prognostic predictor of mortality for critically patients with sepsis (32, 33). In this study, BUN was validated to be an effective predictor of MAKE30 in sepsis patients.

In this study, a straightforward nomogram model was developed and validated to predict the occurrence of MAKE30 in sepsis patients, utilizing variables that are readily available within 24 h of admission. The performance of the designed nomogram was assessed in terms of discrimination, calibration, and clinical application, and it demonstrated satisfactory results after verification. Furthermore, this nomogram model offered valuable insights for guiding treatment decisions in high-risk patients who are likely to experience MAKE30. To illustrate the utilization of the nomogram model, an example is provided: assuming a sepsis patient with an age of 85 years old, a RR of 30 bpm, a lactate of 6 mmol/L, a PaO2 of 60 mmHg, and a BUN of 100 mg/dl. Based on the data presented in Figure 2B, the score for each parameter on the “Points” axis is determined. The aggregate score is then computed by summing the points assigned to all parameters [29 (age) + 16 (RR) + 27 (lactate) + 18 (PaO2) + 33 (BUN) = 123]. This score is indicative of an estimated 88% likelihood of developing MAKE30. Alternatively, one may opt to utilize the online version for a convenient attainment of the identical outcome (Figure 2C, see text footnote 1).

### Limitations

Additionally, it is important to acknowledge the limitations of this study. (1) The retrospective nature of the study introduces the possibility of selection bias, potentially impacting the validity of the results. For example, this study cannot apply to patients with hematological disorders. (2) Certain influential factors such as laboratory indicators (e.g., procalcitonin, albumin, and C-reactive protein), site of infection, drugs, and interventions were not accounted for in this investigation. (3) It is worth noting that the MIMIC database utilized in this study was compiled from medical records spanning a period of two decades, which may introduce variability and potential changes in clinical practices over time. The decision-making process for sepsis management underwent significant changes during this period, which is a perplexing element affecting prediction of MAKE30. Therefore, further multi-center prospective studies with more useful variables added would be needed to verify the results.

## Conclusion

In summary, our study revealed that age, RR, lactate, PaO2, and BUN measured within 24 h of admission are crucial predictors for predicting MAKE30 in sepsis patients. Furthermore, the nomogram model based on these predictors demonstrated excellent performance in terms of discrimination, calibration, and clinical applicability for MAKE30, providing clinicians with valuable information for making timely and personalized management decisions.

## Data availability statement

The raw data supporting the conclusions of this article will be made available by the authors, without undue reservation.

## Ethics statement

Ethical approval was not required for the study involving humans in accordance with the local legislation and institutional requirements. Written informed consent to participate in this study was not required from the participants or the participants’ legal guardians/next of kin in accordance with the national legislation and the institutional requirements.

## Author contributions

XY: Methodology, Writing—original draft. QX: Formal analysis, Investigation, Supervision, Validation, Visualization, Writing—review and editing. YH: Supervision, Validation, Writing—review and editing. TM: Data curation, Formal analysis, Writing—review and editing. JZ: Formal analysis, Investigation, Validation, Writing—review and editing.

## Footnotes

1
https://make30-in-sepsis-patients.shinyapps.io/MAKE30_in_sepsis_patients/
